# Revealing the Microstructural Aspects of the Corrosion Dynamics in Rapidly Solidified Mg-Zn-Y Alloys Using the Acoustic Emission Technique

**DOI:** 10.3390/ma14247828

**Published:** 2021-12-17

**Authors:** Daria Drozdenko, Michiaki Yamasaki, Kristián Máthis, Patrik Dobroň, Shin-ichi Inoue, Yoshihito Kawamura

**Affiliations:** 1 Department of Physics of Materials, Faculty of Mathematics and Physics, Charles University, Ke Karlovu 5, 12116 Prague, Czech Republic; mathis@met.mff.cuni.cz (K.M.); dobronp@karlov.mff.cuni.cz (P.D.); 2 Department of Materials Science, Graduate School of Science and Technology, Kumamoto University, 2-39-1 Kurokami, Chuo-ku, Kumamoto 860-8555, Japan; yamasaki@kumamoto-u.ac.jp; 3 Magnesium Research Center, Kumamoto University, 2-39-1 Kurokami, Chuo-ku, Kumamoto 860-8555, Japan; shinoue7@kumamoto-u.ac.jp (S.-i.I.); rivervil@gpo.kumamoto-u.ac.jp (Y.K.)

**Keywords:** magnesium alloys, rapid solidification, microstructure, corrosion properties, acoustic emission

## Abstract

This work was focused on revealing the relation between the microstructure and corrosion dynamics in dilute Mg_97.94_Zn_0.56_Y_1.5_ (at.%) alloys prepared by the consolidation of rapidly solidified (RS) ribbons. The dynamics of the corrosion were followed by common electrochemical methods and the acoustic emission (AE) technique. AE monitoring offers instantaneous feedback on changes in the dynamics and mode of the corrosion. In contrast, the electrochemical measurements were performed on the specimens, which had already been immersed in the solution for a pre-defined time. Thus, some short-term corrosion processes could remain undiscovered. Obtained results were completed by scanning electron microscopy, including analysis of a cross-section of the corrosion layer. It was shown that the internal strain distribution, the grain morphology, and the distribution of the secondary phases play a significant role in the corrosion. The alloys are characterized by a complex microstructure with elongated worked and dynamically recrystallized α-Mg grains with an average grain size of 900 nm. Moreover, the Zn- and Y-rich stacking faults (SFs) were dispersed in the grain interior. In the alloy consolidated at a lower extrusion speed, the homogeneous internal strain distribution led to uniform corrosion with a rate of 2 mm/year and a low hydrogen release. The consolidation at a higher extrusion speed resulted in the formation of uneven distribution of internal strains with remaining high strain levels in non-recrystallized grains, leading to inhomogeneous growth and breakdown of the corrosion layers. Therefore, homogeneity of the internal strain distribution is of key importance for the uniform formation of a protective layer.

## 1. Introduction

Mg alloys have been used for many engineering applications, where weight saving belongs to crucial issues. Moreover, their usage as bio-implants also has promising potential. In particular, the natural degradation of Mg in biological media is advantageous for the design of temporary implants, which can naturally dissolve in the body after the healing process, and thus secondary surgeries are avoided, bringing benefits for patients by shortened recovery periods and decreased costs. A further important issue is the similarity of the elastic moduli of Mg alloys and cortical bone, respectively. Thus, the stress shielding effect, characteristic of currently used Ti load-bearing implants, is not arising in the case of Mg-based materials [1,2]. However, the low strength and moderate fatigue properties of Mg-based alloys, as well as their hardly controllable degradation rate, still limit their use. In fact, over the last 20 years, many studies have been searching for the Mg alloy with a corrosion rate substantially lower than the intrinsic Mg corrosion rate of 0.3 mm/year as measured by mass-loss in a concentrated chloride solutions [3].

Recently developed Mg alloys with a long-period stacking ordered (LPSO) phase showed improved mechanical properties compared with the common wrought Mg alloys [4,5,6,7]. The type of the LPSO phase is defined by a periodic arrangement of the close-packed atomic layers enriched by rare earth (RE) elements and transition metals (TM) in the Mg lattice [8,9]. The volume fraction, orientation, and distribution of the LPSO phase significantly affect the performance of the material [10,11,12,13,14,15,16,17]. The presence of the fine dynamically recrystallized (DRX) α-Mg grains and large non-DRX grains in extruded Mg-LPSO alloys was found to be highly effective in improving mechanical properties [7]. In addition, the hard LPSO phase provides a fiber-reinforced composite-like strengthening to the alloy. However, from the point of view of corrosion behavior, the LPSO phase is rather problematic, since there is a difference in the electrochemical potential between the α-Mg matrix and the LPSO phase. Experimentally, the difference in the electrochemical potential between the α-Mg matrix and the LPSO phase has been revealed by scanning Kelvin probe force microscopy [18]. As numerous authors have shown, e.g., [19,20], the presence of impurities, alloying elements, and secondary phases are the main reasons for the low corrosion resistance of Mg alloys due to galvanic attack.

It has been shown that ultrafine-grained microstructure can enhance both mechanical and corrosion performance [21,22,23,24,25]. This idea has been applied to Mg-LPSO alloys as well. Several processing techniques, such as powder metallurgy [5,26], extrusion of recycled chips [27,28], equal channel angular pressing [27], rotary swaging technique [29,30] or rapidly solidified (RS) ribbon-consolidation [31,32,33], have been developed to reach the goal of developing the sub-micron grain-sized Mg alloys.

In the case of RS processing of dilute Mg alloys, solute segregated stacking faults (SFs) form dispersedly in the α-Mg grains [32]. Such a morphological change from a massive block-shaped LPSO phase, characteristic for coarse grain material [7,32,34], can lead to an improvement in corrosion resistance without degradation of the mechanical properties [32]. The uniform corrosion mode has been observed in different systems, such as Mg-Ho-Zn [35,36], Mg-Er-Zn [37], or Mg-3Gd-1Zn-0.4Zr [38]. The literature data are ambiguous if the corrosion proceeds along SFs (Mg-Ho-Zn [35,36] and Mg-Er-Zn [37] alloys) or in the α-Mg interior between SFs (Mg-3Gd-1Zn-0.4Zr [38]). The discrepancy is most likely due to the different chemical compositions of the studied materials.

One of the main achievements in the case of the RS ribbon-consolidated Mg-Zn-Y alloys is the random orientation of the grains, which leads to a stochastic distribution of the SFs. Consequently, it rather behaves as a single-phase super-saturated solid solution material, and therefore enhanced corrosion resistance can be achieved. Some preliminary results on the investigation of corrosion of RS ribbon-consolidated Mg-Zn-Y alloys have been reported in [31,39,40]. More information about recent developments on the corrosion behaviors of Mg alloys with SFs or LPSO phase and their beneficial effect is provided in [41,42].

At the same time, from the point of view of biomedical applications, minimizing the alloying elements is a key factor. In our previous work, we optimized the RS ribbon-consolidated Mg-Zn-Y alloy in order to find a good compromise between keeping the alloying content low and maintaining the mechanical properties [43]. We found that two RS ribbon-consolidated Mg_97.94_Zn_0.56_Y_1.5_ alloys can meet this requirement. The yield strength and an elongation of 362 MPa and 18.2%, respectively, were reached for the alloy extruded at a metal flow rate (*mfr*) of 1.9 s^−1^, while the alloy extruded at a *mfr* of 1.4 s^−1^ showed the yield strength of 329 MPa and an elongation of 13.5% [43].

Thus, the present work was focused on the investigation of the corrosion performance of these materials. The RS Mg_97.94_Zn_0.56_Y_1.5_ (at.%) ribbons were consolidated by two different extrusion rates (corresponding to two values of *mfr*) in order to reveal the impact of the microstructure on the corrosion process. As a benchmark material, conventionally cast and extruded specimens having the same composition were used. The corrosion behavior was studied by common electrochemical methods (incl. hydrogen release, electrochemical impedance spectroscopy, cathodic polarization), and an in-situ acoustic emission (AE) technique. The main advantage of AE consists of an instantaneous reaction to the changes in the dynamics and mode of the corrosion. In contrast, electrochemical measurements took place at pre-defined immersion times, i.e., corrosion processes have already been realized for some time, and thus short-term processes could remain undiscovered. Therefore, AE can provide missing information about the development of corrosion processes with high time resolution [44,45,46,47,48]. In addition, propagation of the corrosion layer is studied by backscattered electron (BSE) imaging and grain orientation mapping on the cross-section of the specimens.

## 2. Materials and Methods

The master Mg_97.25_Zn_0.75_Y_2_ (at.%) alloy was prepared by gravity casting in an Ar atmosphere. Afterwards, the master alloy was melted at 750 °C and mixed with pure Mg (99.99%) in order to obtain the Mg_97.94_Zn_0.56_Y_1.5_ alloy, which was used for preparation of RS ribbons using a single roller liquid quenching melt-spinning method [31] at a roll peripheral speed of 42 m/s and a cooling rate of 1.4·10^5^ K/s. Subsequently, the RS ribbons were consolidated by following the procedure: cold compacting into a copper billet (with an applied pressing stress of about 25 MPa), degassing at 523 K for 15 min, and extrusion with extrusion ratio R10 at 623 °K and *mfr* of 1.4 or 1.9 s^−1^, hereafter RS 1.4 or RS 1.9, respectively. Further details about the preparation of the RS ribbon-consolidated Mg-Zn-Y alloy can be found in [31]. The average *mfr* was calculated from extrusion parameters (extrusion ram speed, die angle, billet diameter, and extrusion rate) and represents the average equivalent strain rate, see Appendix B in [49]. To reveal the effect of the microstructure given by the processing on corrosion properties, the alloy with the same composition (Mg_97.94_Zn_0.56_Y_1.5_) was produced by extrusion directly from gravity cast ingot with the same extrusion ratio and *mfr* of 1.9 s^−1^ (hereafter CE 1.9) and subjected to further investigation. The chemical composition of the alloys was determined by inductively coupled plasma (ICP) emission spectrometry and the results are presented in Table 1.

The initial microstructure of the specimens was investigated by scanning electron microscopes (SEM: CrossBeam Auriga, Zeiss, Jena, Germany and JSM-7001F, JEOL, Tokyo, Japan) and transmission electron microscope (TEM: JEM-2000FX, JEOL, Tokyo, Japan). The specimens for the SEM observations were polished down to ¼ µm particle size using diamond paste with intermediate cleaning in an ultrasound bath. As the last step, their surface was treated by ion-milling using the Leica EM RES102 system (Leica, Wetzlar, Germany). Backscattered electron (BSE) imaging, as well as grain orientation mapping using electron back-scattered diffraction (EBSD) technique, were performed on the longitudinal section of the extruded round bar. The EBSD maps were measured with a step size of 75 and 300 nm for RS and CE specimens, respectively, and subsequently processed using TSL OIM Analysis software (version 8, AMETEK, Berwyn, IL, USA). In addition to the evaluation of the grain size, the internal strain distribution was estimated using Kernel Average Misorientation (KAM) mapping [50]. The KAM values were estimated as the average misorientation between the data pixel and the first nearest neighbors with a 5° tolerance angle. The TEM foils were prepared by mechanical polishing and final thinning was performed by Ar ion milling using Gatan PIPS M-691 (AMETEK, Berwyn, IL, USA).

The corrosion rate was evaluated by measuring the volume of evolved hydrogen (H_2_) gas during the immersion tests in 3.5 (*w*/*v*) % NaCl solution saturated with Mg(OH)_2_ for 72 h. The rectangular specimens with a size of 2 × 4 × 8mm^3^ were horizontally immersed in the solution and the evolved H_2_ gas was collected in a burette above the specimens. The instantaneous corrosion rate from the hourly rate of hydrogen gas evolution, *P*_IH_ (mm/y), was calculated using the following Equation:

(1) $$P_{IH}\;=\;353\cdot \frac{M}{\rho \cdot A}\cdot \Delta V_{H},$$

where *M* (g mol^−1^) is the apparent atomic weight of the alloy, *ρ* is density (g/cm^3^), *A* is specimen’s area exposed to the solution, and Δ*V*_H_ (ml/h) is the hourly rate of H_2_ gas evolution. The molar volume at the standard ambient temperature and pressure (SATP, 25 °C and 100 kPa) was estimated to be 24.79 L/mol [18,31]. The average corrosion rate, *P*_Hydrogen_ (mm/y), was evaluated from the total volume of H_2_ gas, *V*_H_ (mL), evolved for the total immersion time, *t* (h):

(2) $$P_{Hydrogen}\;=\;353\cdot \frac{M}{\rho \cdot A}\cdot \frac{V_{H}}{t}.$$

In addition, the corrosion rates were also estimated using electrochemical impedance spectroscopy (EIS). A standard three-electrode setup was used for the electrochemical experiments with an Ag/AgCl saturated KCl reference electrode, a specimen operating as a working electrode, and a platinum plate serving as a counter electrode. The diameter of the specimen’s surface exposed to the solution was 7 mm. For a proper evaluation and fitting of the data, cathodic polarization was performed after 72 h of immersion. The EIS and cathodic polarization measurements were performed with a scan rate of 10 mV/min using the PARSTAT 3000 potentiostat. The obtained Nyquist plots were fitted using models of equivalent electrical circuits described in [51,52,53]. The instantaneous corrosion rate from EIS, *P*_EIS_ (mm/y), was determined using the corrosion current density, *i*_*corr*_ (A/cm^2^), as follows:

(3) $$P_{EIS}\;=\;1634\cdot \frac{i_{corr}\cdot M}{\rho }.$$

The corrosion current density, *i*_*corr*/*EIS*_ (A/cm^2^), can be determined from the polarization resistance, *R*_p_ (Ohm·cm^2^), using the Stern-Geary Equation [54]:

(4) $$i_{corr/EIS}\;=\;\frac{\beta_{c}\beta_{a}}{2.303\cdot R_{p}\cdot \left(\beta_{c}\;+\;\beta_{a}\right)}=\frac{B}{R_{p}}\;,$$

where *β*_c_ (V/decade) and *β*_a_ are the cathodic and anodic Tafel slopes, respectively. *B* is the Stern-Geary coefficient. The Levenberg-Marquardt method [55,56] was used to fit the measured cathodic polarization curves from *E*_ocp_ +20 mV to +250 mV using the following Equation (5):

(5) $$i\;=\;i_{corr}\left(10^{\left(E\;-\;E_{corr}\right)/\beta_{a}}\;+\;10^{-\left(E\;-\;E_{corr}\right)/\beta_{c}}\right),$$

which produced reasonable fit values of *β*_c_ and *β*_a_.

In order to obtain statistically relevant data, all corrosion tests were performed for several specimens (from 3 to 9) under each condition. To observe the microstructure of the corrosion layers of the immersed surface at different stages of immersion tests, the cross-sections of the specimens were ion-milled using JEOL Cross Section Polisher SM-09010 (JEOL, Tokyo, Japan). Furthermore, the microstructure was studied in detail by SEM, including BSE imaging and EBSD mapping.

In order to record the AE response during immersion, an acrylic glass cell with a hole for plugging in the specimens was used. An AE sensor was attached to the outside side of the specimen with the help of a screw. More information about the setup can be found in [44]. The specimens were exposed at room temperature to a 3.5 (*w*/*v*) % NaCl solution saturated with Mg(OH)_2_. To monitor AE activity, a computer-controlled Vallen device (Wolfratshausen, Germany) with continuous storage of AE signals and a 2 MHz sampling frequency was used. A piezoelectric PAC (Physical Acoustics Corporation, West Windsor Township, NJ, USA) nano-30 AE sensor was used and the AE signal was pre-amplified by 34 dB using a Vallen AEP5H preamplifier. The possible noise coming from the electrical circuit and occurring during the polarization test was eliminated by a ceramic surface of the AE sensor. The AE signal was evaluated using a standard threshold-based evaluation method (ASTM E1316). Particularly, the AE count rate (∆NC/∆t)—the count number per time unit [57] at a given threshold voltage level—and a cumulative number of counts (Cumulative Counts) were calculated using a threshold level of 27.2 dB, which is level above the background noise.

## 3. Results and Discussion

**Figure 1:**
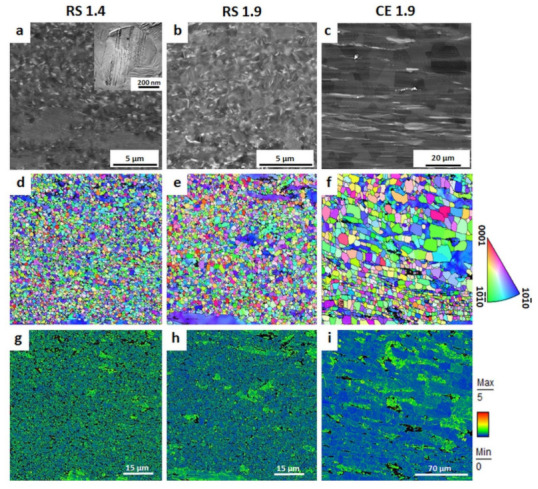
Initial microstructure of RS ribbon-consolidated alloys (RS 1.4 and RS 1.9) and cast-extruded alloy (CE 1.9) obtained by SEM: (**a**–**c**) BSE images, (**d**–**f**) orientation mapping, and (**g**–**i**) KAM mapping. All micrographs are taken on a longitudinal section with ED oriented horizontally. The color designation is in ED, i.e., original EBSD maps are rotated toward ED.

The *initial microstructures* of the RS ribbon-consolidated alloys (RS 1.4 and RS 1.9) together with a microstructure of the cast-extruded alloy (CE 1.9) are presented in Figure 1. The white contrast in BSE images (Figure 1a–c) corresponds to the Zn and Y enrichment, while the grey contrast corresponds to the α-Mg phase. The microstructure of the RS ribbon-consolidated alloys was characterized as bimodal with fine DRX grains and non-DRX grains elongated along the extrusion direction (ED). Information about the orientation and size of the grains was provided by EBSD maps (Figure 1d–f), where the color code triangle corresponds to the orientation of the hcp α-Mg phase. For all EBSD maps, a condition of confidence index (CI) higher than 0.1 was applied. Therefore, the black areas in maps correspond to the regions with CI < 0.1 (i.e., regions where the obtained Kikuchi diffraction patterns cannot be properly indexed). Those areas can be either highly deformed regions or there is a large concentration of SFs, which may distort diffraction patterns of the α-Mg phase. The original EBSD maps were reoriented to adjust colors designation with respect to ED. Despite the overall random texture, it is obvious that elongated non-DRX grains had their <10–10> direction oriented parallel to ED. This alignment can be explained by the lattice rotation due to the activation of basal and prismatic slip with the <11–20> slip direction during the hot extrusion process [58]. The elongated grains having <10–10> axis parallel to ED have previously been observed in wrought Mg alloys [59], as well as in Mg-LPSO alloys [13,34]. The average grain size of RS 1.4 and RS 1.9 was estimated to be (837 ± 171) and (912 ± 202) nm, respectively. Moreover, rapid solidification of the dilute Mg-Zn-Y alloy led to the formation of the solute segregated SFs dispersedly distributed in the DRX grains (Figure 1a,b) rather than the block-shaped LPSO phase elongated along ED, as it is in the case of extruded alloys. As can be seen in the representative TEM micrographs (extra image in Figure 1a) taken with the <0001> direction perpendicular to the beam, the SFs were oriented parallel to the basal plane in the α-Mg phase, i.e., enriched layers of Zn and Y are formed in basal planes. Results of X-ray diffraction measurements and TEM observations of RS ribbon consolidated Mg_97.94_Zn_0.56_Y_1.5_ alloys reported in our previous works [39,43] did not indicate any other intermetallic compounds or inclusions. More details about the microstructure and texture of RS ribbon-consolidated Mg-Zn-Y(-Al) alloys can be found in [32,39,43]. On the contrary, the extrusion of the cast material results in the formation of a microstructure with massive LPSO phase laths (as a second phase) and α-Mg grains with an average grain size of 9 µm, Figure 1c. Similar microstructures have been observed in other Mg-LPSO alloys [7,11,12,14,17,32,34,60,61,62].

The *KAM maps* (Figure 1g–i) indicated a homogeneous distribution of internal strain in RS 1.4 with relatively high values of KAM in both DRX and non-DRX grains. Herein, the term “internal strain” is used in the meaning of a total internal strain including plastic and elastic components. At the same time, RS 1.9 was characterized by a bimodal microstructure with a variation in internal strain: DRX grains were characterized by significantly lower KAM values compared with those in non-DRX worked grains. An uneven distribution of KAM angles in the case of RS 1.9 is given by applying a higher extrusion speed compared with the one for RS 1.4. During extrusion at *mfr* of 1.9 s^−1^, the recrystallization temperature was easily reached by higher friction, which led to partial recovery processes in the microstructure and a formation of a specific bimodal microstructure with variation in internal strain. Segregation of solute atoms of Y and Zn on grain boundaries limited the recrystallization of larger elongated grains, and high internal strain remained inside non-DRX grains. In the case of CE 1.9, the distribution of internal strain (Figure 1i) was also inhomogeneous with localization of high KAM values in some grains. Those grains were also characterized by a gradient of the orientation axis (blue grains with a color gradient on the right side of the map in Figure 1f) given by deformation introduced during extrusion. Therefore, despite the homogeneous size distribution in CE 1.9, those grains were supposed to be non-DRX grains.

**Figure 2:**
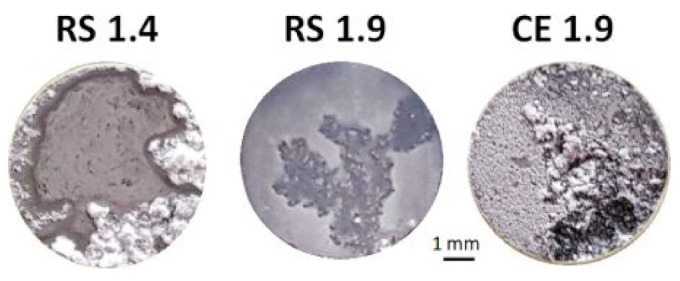
Surface morphology of RS 1.4, RS 1.9, and CE 1.9 after immersion in 3.5 (*w*/*v*) % NaCl solution for 72 h.

The surface morphology of the specimens after 72 h of immersion indicated that both RS alloys have better corrosion resistance compared with CE 1.9, Figure 2. The extent of corrosion on the surface of CE 1.9 was significantly larger than that for RS specimens.

**Figure 3:**
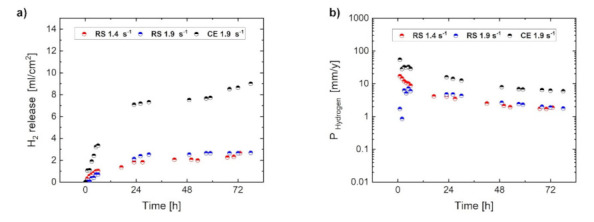
Evolution of H_2_ release (**a**) and corrosion rate (P_Hydrogen_) (**b**) for RS 1.4, RS 1.9, and CE 1.9 specimens during immersion in 3.5 (*w*/*v*) % NaCl solution.

The *evolutions of H_2_ release* and the calculated corrosion rate (P_Hydrogen_) of the RS ribbon-consolidated and cast-extruded specimens during immersion in 3.5 (*w*/*v*) % NaCl solution saturated with Mg(OH)_2_ are represented in Figure 3. It is obvious that volumes of the H_2_ release and the related corrosion rates of both RS 1.4 and RS 1.9 alloys were lower than that of the CE 1.9 alloy. Nevertheless, the overall values of the corrosion rates were higher than the intrinsic Mg corrosion rate of 0.3 mm/year revealed by mass-loss in a concentrated chloride solution [3]. However, obtained results of the corrosion rate for RS material, being lower compared to those of other Mg alloys, provide a motivation for further development of this type of alloy (using a low amount of alloying elements and the application of a rapid solidification technique). All three specimens were characterized by the rapid evolution of H_2_ gas at the beginning of the immersion test. During the first 24 h of immersion, H_2_ release values reached a certain level at which they remained relatively stable for the next 48 h. Thus, it can be assumed that during the first 24 h, a basic corrosion layer is formed, which temporarily protects the material.

**Figure 4:**
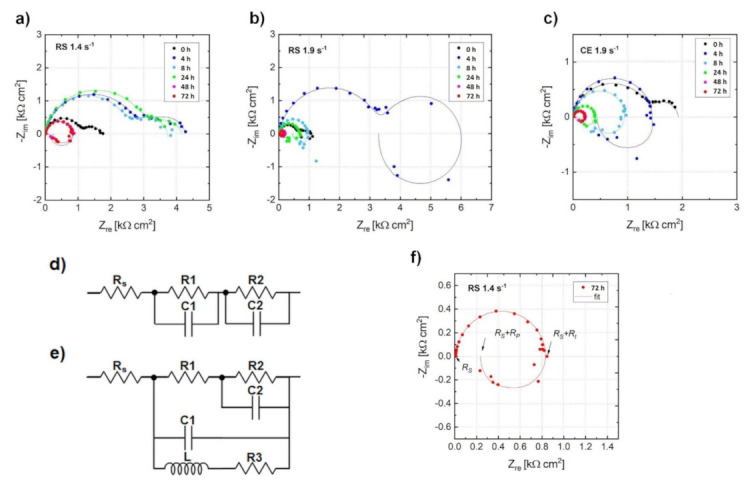
Fitted Nyquist plots for the (**a**) RS 1.4, (**b**) RS 1.9, and (**c**) CE 1.9 alloys immersed in 3.5 *w*/*v* % NaCl solution saturated with Mg(OH)_2_, using the equivalent circuits (**d**,**e**). The typical Nyquist plot (**f**) for the RS 1.4 immersed in a 3.5 *w*/*v* % NaCl solution saturated with Mg(OH)_2_ for 72 h with respect to the equivalent circuit shown in (**e**).

**Figure 5:**
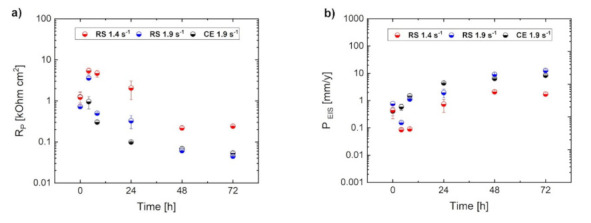
Corrosion resistance (R_p_) (**a**) and corrosion rate (P_EIS_) (**b**) for RS 1.4, RS 1.9 and CE 1.9 specimens estimated from electrochemical impedance spectroscopy (EIS) during immersion in 3.5 (*w*/*v*) % NaCl solution.

In addition, the corrosion rate, *P_EIS_*, was estimated from *electrochemical impedance spectroscopy (EIS)*. Figure 4 shows Nyquist plots for investigated alloys immersed in a 3.5 *w*/*v* % NaCl solution saturated with Mg(OH)_2_ for 72 h and equivalent circuits used in this study. The Nyquist plots for the RS 1.4 specimen (Figure 4a) display only the capacitive loops up to 24 h of immersion; the plots can be simulated using an equivalent circuit consisting of a series of two parallel resistance and constant phase element networks as shown in Figure 4d. After 48 h of immersion, an inductive loop showed up; in this case, the plots can be simulated using another equivalent circuit considering a physical model based on the adsorbed intermediates in the Mg corrosion reaction as shown in Figure 4e. On the other hand, the Nyquist plots for RS 1.9 and CE 1.9 displayed capacitive loops at high frequencies and an inductive loop at low frequencies already after 4 h of immersion, as shown in Figure 4b,c. This study defined the charge-transfer resistance (*R*_t_) as the value corresponding to *Z*_re_ when *Z*_im_ is zero at intermediate frequencies. The polarization resistance (*R*_p_) was evaluated as the real component of the impedance by extrapolating the low-frequency EIS data to the limit when the frequency reached zero. An example of fitting the Nyquist plot for the RS 1.4 immersed in 3.5 *w*/*v* % NaCl solution saturated with Mg(OH)_2_ for 72 h with respect to the equivalent circuit and definition of polarization resistance is shown in Figure 4f. The changes in R_p_ as functions of immersion time are shown in Figure 5a. The value of R_t_ were plotted instead of R_p_ when the inductive loop did not appear. Figure 5b represents the instantaneous corrosion rates estimated from EIS-derived *R*_p_ values. One can conclude that the corrosion rates (0.8–10 mm/year range) estimated from H_2_ release and EIS measurements are in a good agreement with the observations by other authors, performed on similar alloy systems [3,32,33,39,40,63]. Nevertheless, the EIS measurements showed a different evolution of the corrosion process for the particular specimens. It is obvious from Figure 5a that the corrosion resistance, *R_p_*, estimated from EIS measurements for RS 1.4, was higher than that of RS 1.9 and CE 1.9, which exhibited comparable values. This means that the EIS measurement did not provide comprehensive information about the corrosion and a deeper analysis is required, particularly including the study of the corrosion layer evolution.

**Figure 6:**
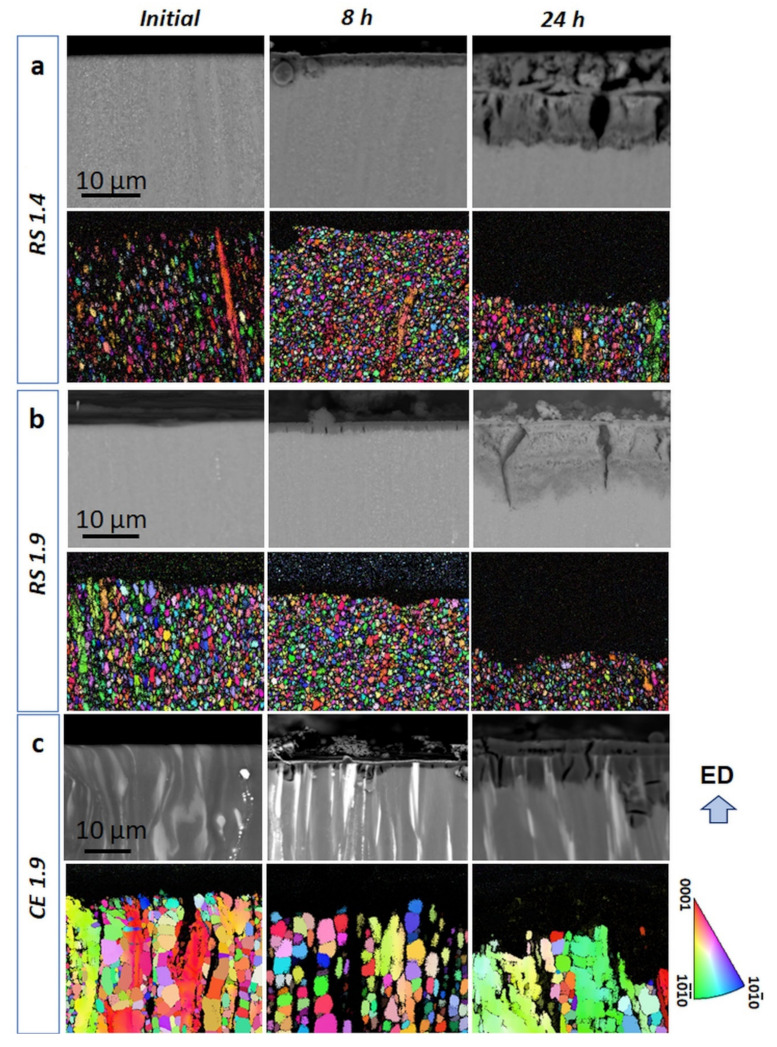
Development of the corrosion layer (cross-section view) formed on the surface of (**a**) RS 1.4, (**b**) RS 1.9, and (**c**) CE 1.9 specimens during immersion in 3.5 (*w*/*v*) % NaCl solution. The BSE images demonstrate the different evolution of the corrosion layers for the particular specimens. It is obvious from the comparison of the EBSD maps and BSE images that the highly-strained non-DRX grains and α-Mg phase—LPSO-phase interface are the preferential sties for corrosion.

Therefore, the cross-section of the corrosion layer after immersion for 8 and 24 h was investigated using BSE imaging and EBSD mapping (see Figure 6). Such a blend of composition- (BSE) and grain morphology related information can help to find the link between the microstructure and corrosion behavior. It can be seen in BSE images (second column in Figure 6a,b) that after 8 h of immersion, a thin corrosion layer was formed on the surface of the RS 1.4 and RS 1.9 specimens, while the surface layer was significantly thicker for the CE 1.9 specimen (cf. Figure 6c). After 24 h of immersion (third column in Figure 6), the thickness of the corrosion layer increased for all three specimens (note that the real thickness of the corrosion layer for CE 1.9 is supposed to be larger than that shown, since part of the layer is continuously exfoliating from the specimen surface). Nevertheless, the average thickness of the layer formed on the surface of RS 1.4 was the same throughout the entire specimen surface, indicating a homogeneous formation and growth of the layer. In the case of the RS 1.9 specimen, the thickness of the layer varied and, in some places, the corrosion propagated deeper into the specimen volume by the LPSO lathes (cf. non-indexed black areas in the EBSD image in Figure 6b), which are supposed to be weak sites for corrosion in this specimen.

This behavior was even more pronounced for the CE 1.9 specimen. Homogeneous proceeding of the corrosion layer (Figure 6a) resulting in high corrosion resistance (*R_p_*, Figure 5) in the case of RS 1.4 can be related to ultra-fine-grained microstructure with a homogenous distribution of internal strain (Figure 1g). This allowed an even propagation of the corrosion front from the surface to the material’s interior, leaving behind a relatively flat surface faced to the solution. The inhomogeneity of the corrosion layer (Figure 6b,c) resulting in low corrosion resistance (*R_p_*, Figure 5) of RS 1.9 and CE 1.9 can also be linked to the inhomogeneity of microstructure, particularly to the presence of localized concentrations of internal strains in non-DRX grains (see Figure 1h,i). During immersion test, these highly strained non-DRX grains became weak sites for corrosion resistance, leading to inhomogeneous corrosion propagation [64]. In the CE 1.9 specimen, the massive LPSO-phase laths acted as cathodic sites, causing severe galvanic dissolution of α-Mg matrix (see Figure 6c). Therefore, severe local corrosion attacks occurred in the α-Mg phase next to LPSO-phase laths. However, in the RS ribbon-consolidated alloys without the LPSO laths, no local anodic dissolutions were observed. In this case, due to suppressing the effect of potential difference, the internal strain inhomogeneity in the microstructure became important. However, globally, the influence of the potential difference between LPSO and α-Mg phase on the corrosion behavior of Mg-Zn-Y alloys is supposed to be stronger than that of grain size and internal strain. The results of a full-scale investigation of morphology and characterizations of the corrosion products formed on RS Mg-Zn-Y alloys will be reported elsewhere in the near future.

**Figure 7:**
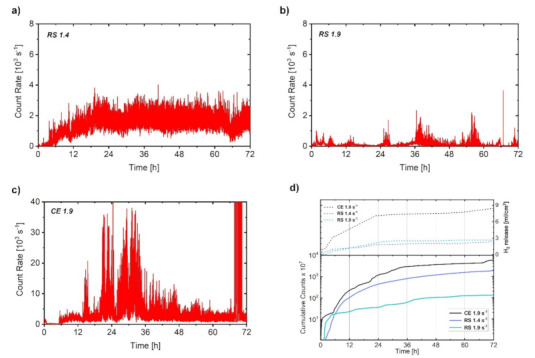
Analysis of the AE response: AE count rate (**a**–**c** for RS 1.4, RS 1.9, CE 1.9, respectively) and (**d**) cumulative counts correlated with the evolution of H_2_ release during immersion of in a 3.5 (*w*/*v*) % NaCl solution. Please note the different scale for the AE count rate in (**a**–**c**).

The *AE response* was monitored continuously during the first 72 h (3 days) of immersion (Figure 7). The main advantage of the AE technique in corrosion monitoring is the possibility to reveal some transient effects such as the rapid breakdown of the corrosion layer, which is not visible from the electrochemical corrosion measurements due to their low time resolution. We used the AE counts for the characterization of the corrosion process. The cumulative value of AE counts allows one to directly compare the corrosion of the particular specimen by its AE activity (in the sense that more AE is given by more intensive corrosion). The AE count rate (number of counts per second) allows following the above-mentioned transient effects, i.e., dynamics of the processes.

The AE count rate for RS 1.4 was low in the first couple of hours and then gradually increased during the first 24 h. Afterwards, the AE response was stable and it oscillated around a constant level until the end of the test. In the case of the RS 1.9 specimen, the AE count rate had an impulsive character: high AE activity periods were followed by almost quiet intervals. The overall average values of the count rate for RS 1.9 were lower than those for the RS 1.4 specimen, what can be given by corrosion in DRX grains with low internal strain. The magnitude of the AE activity for the CE 1.9 specimen was significantly higher compared with those for RS 1.4 and RS 1.9 (please note the different scale of the AE count rate plots in Figure 7) and its impulsive character was similar to that for RS 1.9. The high peak intensity of the AE count rate in the case of CE 1.9 can be associated with the high H_2_ release (Figure 2), which can be substantiated by promoted corrosion due to the presence of the LPSO laths [32,39]. The impulsive character of the AE response for RS 1.9 and CE 1.9 (Figure 7b,c) can be related to an inhomogeneous formation of the corrosion layer due to variation in the strain distribution (Figure 1h,i). In contrast, in the case of RS 1.4, a homogeneous distribution of internal strain with relatively high average values (Figure 1g) resulted in a homogeneous growth of the corrosion layer. The corrosion process, in this case, is supposed to be intense and time-stable as indicated by the stable AE response, Figure 7a.

The developments of cumulative counts showed its rapid increase during the first 6 h of immersion for all three investigated alloys. Afterward, values of cumulative counts continued to gradually increase for the next 18 h and 30 h in the case of RS 1.4 and CE 1.9, respectively, which corresponds to the evolution of the AE count rate. After reaching the saturated level, cumulative counts for both specimens remained at the same level until the end of the test. In contrast, the values of cumulative counts for RS 1.9 increased rather slowly after 6 h of immersion and after 72 h of immersion reached one order lower values than those for RS 1.4 and CE 1.9. This is given by overall low intensity of the signal for RS 1.9 compared with those for RS 1.4 and CE 1.9 (Figure 7b vs. Figure 7a,c).

It is also obvious that the general tendency of the time evolution of the cumulative counts was in good agreement with the evolution of H_2_ release, Figure 7d. Particularly, both time dependences showed a rapid increase in values during the first 6 h followed by a slow increase up to 24 h, and after reaching the saturated level, both dependences persisted with a linear response until the end of the test (72 h). However, the variation in character (stable or impulsive) and intensity of the AE response indicated a significant difference in the corrosion film formation for the particular RS materials, which is not seen in common corrosion tests (a similar H_2_ release was revealed for both RS materials).

In particular, in the case of RS 1.9, the low-intensity AE, indicating a slow corrosion layer propagation (in low-strained grains), was overlapped by strong burst-type signals, which manifested intense corrosion layer cracking. Therefore, the overall formation of the corrosion layer in this alloy is rather non-uniform. In contrast, in RS 1.4, the average AE count rate was higher, owing to the promoted corrosion layer formation, but the AE bursts were not present since the corrosion was uniform due to the homogeneous distribution of internal strain.

Therefore, despite the Mg_97.44_Zn_0.56_Y_1.5_ alloy extruded at *mfr* of 1.9 s^−1^ showing high yield strength and moderate elongation (362 MPa and 18.2%, respectively), given by a bimodal microstructure with variation in grain size and internal strain distribution, an uneven corrosion layer development is undesired. In contrast, the slightly lower mechanical performance (329 MPa and 13.5%, respectively) of the Mg_97.44_Zn_0.56_Y_1.5_ alloy extruded with *mfr* of 1.4 s^−1^ is compensated by a uniform formation of the corrosion layer, leading to a higher application potential.

## 4. Conclusions

The relationship between the microstructure features and corrosion performance of the rapidly solidified (RS) ribbon-consolidated Mg_97.44_Zn_0.56_Y_1.5_ alloy extruded with a metal flow rate (*mfr*) of 1.4 and 1.9 s^−1^ was investigated. The results were compared with the performance of the conventionally cast-extruded alloy having the same composition but a larger grain size and fraction of LPSO phase. The experimental approach included a combination of electrochemical techniques with acoustic emission (AE) measurement and scanning electron microscopy. On the basis of the experimental findings, the following conclusions can be drawn:

- Material processing significantly influences the microstructure. The application of the rapid solidification technique leads to the formation of ultrafine-grained (700–1200 nm) material with the solute segregated stacking faults dispersedly distributed in the grain interior. The rate of consolidation extrusion influences the dynamic recrystallization and leads to a difference in the internal strain distribution. In contrast, large LPSO laths form in the cast-extruded alloys.
- The RS ribbon-consolidated alloy extruded with a metal flow rate of 1.4 s^−1^ is characterized by a homogeneous distribution of the internal strain. At a higher metal flow rate (1.9 s^−1^), the distribution of the internal strain becomes uneven with higher values in worked non-dynamically recrystallized (DRX) grains than those in DRX grains.
- The presence of LPSO laths in the extruded alloy leads to severe anodic dissolution in the α-Mg grains next to the LPSO phase. In the RS alloys without the LPSO laths and, thus, suppressing the effect of potential difference, the strain inhomogeneity in the microstructure became more important for corrosion behavior.
- An uneven distribution of the internal strain results in inhomogeneous development of the corrosion layer even in ultra-fine-grained material. Despite similar values of H_2_ release for RS materials, the instantaneous corrosion rate estimated from electrochemical measurements for the RS 1.9 specimen is slightly higher than that for the RS 1.4 specimen. This is given by the presence of strain inhomogeneity in the microstructure of RS 1.9, while the RS 1.4 specimen with even internal strain distribution exhibits a homogeneous formation and growth of the corrosion layer.
- The AE method was found to be capable of revealing the dynamics of the corrosion process. The uniform corrosion of the RS 1.4 was evidenced by a steady-level AE signal. In contrast, the AE response of the RS 1.9 and CE 1.9 specimens has an impulsive character, indicating inhomogeneous formation and breakdown of the corrosion layer.

## Figures and Tables

**Table 1:** Chemical compositions of the master alloy.

Alloy	Mg	Zn	Y	Fe	Co	Ni	Cu
	(at.%)	(at.%)	(at.%)	(ppm)	(ppm)	(ppm)	(ppm)
Mg_97.94_Zn_0.56_Y_1.5_	Bal.	0.56	1.5	11	–	2	18

## Data Availability

The data are available upon reasonable request from the corresponding author, D.D.
